# Relationship between Physiological Resorption of Primary Molars with Its Permanent Successors, Dental Age and Chronological Age

**DOI:** 10.3390/children9070941

**Published:** 2022-06-23

**Authors:** Antonia M. Caleya, Nuria E. Gallardo, Gonzalo Feijoo, M. Rosa Mourelle-Martínez, Andrea Martín-Vacas, Myriam Maroto

**Affiliations:** 1 Department of Dental Clinical Specialties, Faculty of Dentistry, Complutense University of Madrid, 28040 Madrid, Spain; amcaleya@ucm.es (A.M.C.); negallar@ucm.es (N.E.G.); gfeijoo@ucm.es (G.F.); mrmourel@ucm.es (M.R.M.-M.); mmarotoe@ucm.es (M.M.); 2 Faculty of Dentistry, Alfonso X El Sabio University, 28691 Villanueva de la Cañada, Spain

**Keywords:** dental root resorption, paediatric dentistry, root resorption, dental age, crown-to-root ratio

## Abstract

The aim of the present study was to analyse root resorption of the primary mandibular molars and their relationship with their permanent successors and the age of the patient. Methods: The sample consisted of 408 digital panoramic radiographs. The mesial and distal crown-to-root ratios (CRR) of #74 and #75 were calculated by dividing the measures of the length of each root by its coronal height. The Demirjian formation stage of the premolar was established, and dental age was determined. A descriptive and statistical analysis was performed using SPSS to determine the correlation between the variables (Pearson’s correlation coefficient) and to identify the differences between them (Student’s *t*-test), with a confidence level of 95%. Results: 723 molars were measured, and tables of CRR depending on dental and chronological age were obtained. The CRR decreased with increasing dental and chronological age, but not uniformly. The CRR of #74 and #75 decreased slightly when the successor premolar was in the initial stages of formation. Gender differences were obtained with respect to chronological age, mainly in girls, because the root resorption of #74 was always more advanced, and the formation of the #34 more advanced. Conclusions: Root resorption of the molar is slight and progressive when the successor premolar begins formation until stage D, and becomes higher starting at stage E. It is possible to determine the state of the child’s maturation and the CRR according to dental and chronological age.

## 1. Introduction

Root resorption of primary teeth is a physiological process whose aetiology is not yet fully known [1,2]. Permanent teeth play a very important role, as they exert pressure that causes the release of monocytes, which become osteoclasts and odontoclasts, initiating resorption [3,4,5]. Therefore, other factors are involved, such as hereditary, endocrine, nutritional, and local factors (inflammatory processes, vascularization at the site of resorption, and occlusal trauma) [1,2,3,4,6,7,8]. However, root resorption also occurs without the permanent successor, although it is usually delayed, and the primary molar may present infraocclusion [9,10,11,12].

Very few studies have assessed this physiological event. Almost all of the studies are longitudinal, and several have been carried out in animals. The first author to publish data on the subject was Elizabeth A. Fanning in 1961, who established how the primary teeth are resorbed in seven stages [11]. Years later, Bjerklin et al. published another longitudinal study about resorption of second primary molars without a permanent successor. Their method established six stages, and explained resorption of each root independently [9]. Regarding the relationship between the roots of primary molars and the development of premolar successors, in 1973, Haavikko studied the relationship between resorption of primary teeth and formation of their permanent successors in a sample of 885 orthopantomographies from children between 2 and 13 years old. They observed that premolars have between half and three-quarters of their crown formed when root resorption of primary molars begins, and the permanent successors have formed between one-half and three-quarters of their roots when the resorption of the primary teeth is complete [13]. The germ of the lower premolar is ideally positioned between the roots of the primary lower molar, which guides its eruptive path. At an early stage of development, it is very common to find a distal inclination in this germ, but it moves from an inclined to a more vertical position during growth [14,15]. However, an abnormal position of the germ and an altered eruptive trajectory can lead to asymmetric resorption of primary molar roots [2]. The aim of the study was to measure root resorption in primary molars at a specific time, and to relate it to premolar successor development and with dental and chronological age of the patient.

## 2. Materials and Methods

A retrospective cross-sectional radiographic study was carried out according to the Declaration of Helsinki’s ethical principles for medical research involving human subjects. Informed consent from patients and/or their legal guardians was obtained from all the children before conducting the research.

We obtained the list of all the panoramic radiographs of active patients in the master’s degree in paediatric dentistry. The period of time that the study lasted was two academic years. The initial number of orthopantomographs analysed was 1974, and after applying the inclusion and exclusion criteria, the sample was comprised of 408 digital panoramic radiographs (225 boys and 183 girls). All radiographs were taken at the School of Dentistry, Complutense University of Madrid, Spain. All the records were obtained with the same X-ray machine (Instrumentarium Orthopantomograph^®^ OP30) and parameters, with a known magnification of 25%, being taken in natural head position and by the same person. The records were requested for diagnostic purposes unrelated to this study.

The inclusion criteria for selecting the sample were orthopantomographs from patients between 4 and 12 years old. At least one of the two primary mandibular left molars must have been present on the X-ray. The exclusion criteria were as follows: patients with systemic or syndromic disease or congenital maxillofacial malformation, X-rays with dental alterations that could affect odontogenesis, agenesis of a permanent mandibular left tooth, and other alterations in the primary mandibular left first and/or second molars, such as pathological wear, dental trauma, extensive caries, large reconstructions, pathology and/or pulpal treatment, local morphological alterations, and atypical root resorptions. In addition, radiographs with ectopic eruptions, morphological alterations, or premolar successors in a rotated position were excluded.

The measurement was made using conventionally printed radiographs, with a tabletop negatoscope and with artificial light (blinds lowered) to avoid light variations. In each session, a maximum of 30 radiographs were evaluated to avoid operator fatigue, and these were measured with the naked eye (without magnifications) with a precision calibre with fine points (Dentaurum 042-751 Zürcher model, precision of one tenth of a millimetre). Two pre-calibrated examiners analysed the radiographs, the first being the main researcher, and the other a second examiner that served as support to the main examiner, analysing samples in which the first examiner had doubts in order to reach an agreement. The main researcher evaluated all the of samples, and a month later examined nearly 15% of the sample again as randomly selected orthopantomographs (n = 37) in order to obtain inter-examinator concordance. A second examiner analysed nearly 15% of the sample as randomly selected radiographs (n = 36), and calculated the CRR in 202 first and 232 s primary molars to carry out the inter-examinator concordance.

**Figure 1:**
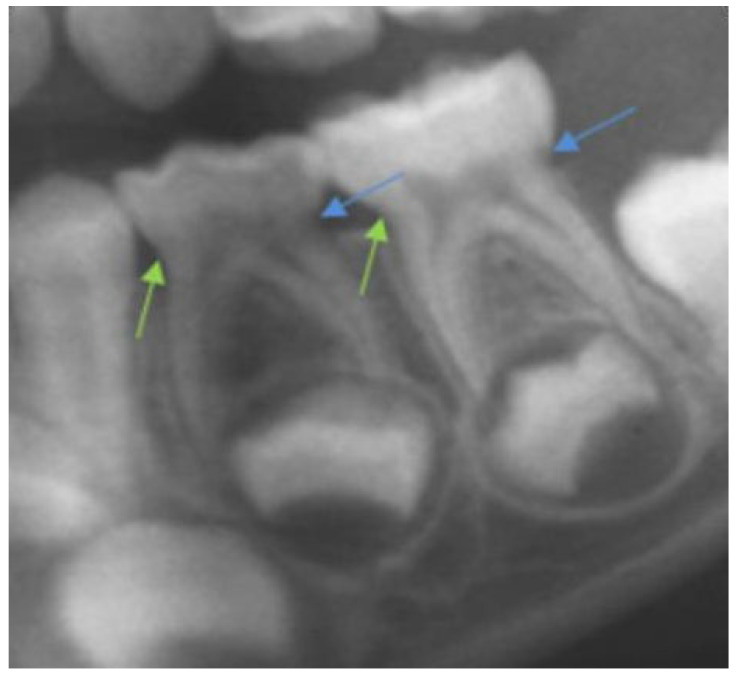
Magnification detail of an orthopantomograph, in which the reference of the dentin–enamel junction is observed in mesial (green arrows) and distal (blue arrows).

We based our study on the methods proposed by Black [16] and Ash [17] with modifications, as some anatomical points of the measurements indicated by these authors are not visible radiographically. The following parameters were obtained:

- Crown height was calculated as the distance between the line that links the mesial and distal cemento-enamel junctions and the highest point of the occlusal surface of the molar (Figure 1).
- Mesial root length was calculated as the distance between the mesial cemento-enamel junction and the most apical point of the mesial root.
- Distal radicular length was calculated in the same way as mesial root length, but in the distal molar area (Figure 1).
- Mesial and distal crown-to-root ratios (CRR-m and CRR-d, respectively) were calculated by dividing the length of each root by its coronal height.

To determine the stage of development of the successor premolars, the method proposed by Demirjian [18,19] was used, in which he establishes eight stages of development (stages A–H) described and drawn by means of diagrams. For the determination of dental age, the method described by Demirjian was also followed, determining the stage of development of the seven left mandibular teeth and using the author’s conversion tables that correspond to the 50th percentile maturation data to obtain dental age [18,19]. Chronological age was calculated using the patient’s date of birth and the date of taking the orthopantomography in years, by subtracting the date of the radiographic record from the date of birth.

The analysis of the results was performed using the SPSS version 19.0 for Windows software (SPSS Inc., Chicago, IL, USA). The intraclass correlation coefficient was used to evaluate intra- and inter-examiner concordance or agreement. A descriptive and statistical analysis was performed. The Pearson’s correlation coefficient analysis was used to determine the correlation between chronologic age and state of development of #74 and #75, and the correlation between the stage of formation of #74 and #75. The Student’s t-test was carried out to identify the differences with respect to the variables studied (the CRR of the successors premolar and chronological age, the stage of development of the premolars and chronological age, the CRR of the mesial and distal, and the development of the permanent successors). A *p*-value ≤ 0.05 (95% significance) was considered significant.

## 3. Results

A total of 723 primary mandibular left molars (322 first molars and 401 s molars) were measured. The sex distribution of the measured molars was homogeneous, with 54.96% boys and 45.04% girls for the first primary molar (tooth #74), and 55.11% boys and 44.89% girls for the second primary molar (tooth #75). The distribution of the sample in each age group was not homogeneous, since the largest number of children were in the chronological age groups of 7–9 years of age, which means that for the extreme values of age (especially for 11- and 12-year-olds) the data should be interpreted with caution. Intra-examiner agreement was excellent for all parameters, while inter-examiner agreement was excellent for all measures except for the distal root length of #75, which was fair.

**Figure 2:**
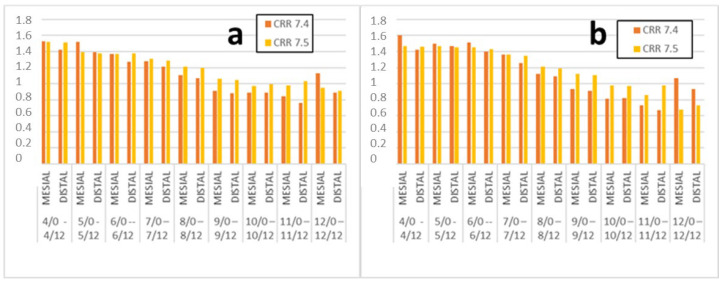
The CRR of #74 and #75, classified according to chronological age (**a**) and dental age (**b**).

**Figure 3:**
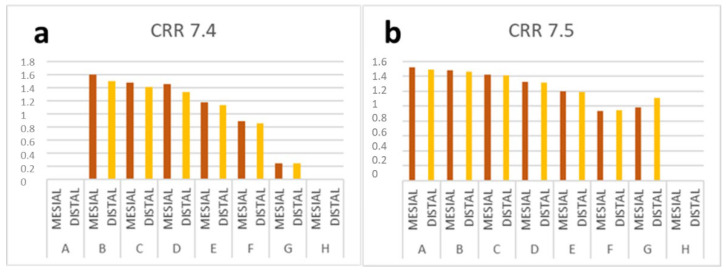
The CRR of #74 and #75, according to the stage of development of #34 (**a**) and #35 (**b**).

The CRR decreased with increasing dental and chronological age but resorption was not a continuous process (Figure 2, Table A1 and Table A2 in Appendix A). The roots of the first primary molar (#74) had minimal resorption when the premolar was in stages B–D of development. The mesial root was the longest until stage G when the two roots were the same length (Figure 2, Table A3 in Appendix A). The roots of the second primary molar (#75) had minimal resorption when the premolar was in stages A–D. The mesial root of #75 was longer than the distal root at the beginning, but both roots were of equal length beginning at stage F (Figure 3, Table A3 in Appendix A).

Differences between sexes were analysed, determining that the crown height of #75 was significantly higher in boys than in girls (*p* = 0.014). In addition, the differences with respect to gender with chronological age were studied, revealing that the main differences between the sexes are produced in the root resorption of #74, being always more advanced in girls, and in the formation of the first premolar (tooth #34), being always greater in girls than in boys, as shown in the following:

- The developmental stage of #34 was greater in girls than in boys of 5 years old (*p* < 0.05)
- The length of the mesial and distal roots, and the CRR-m and CRR-d of #74, was greater in boys of 7 years old than in girls (*p* < 0.05)
- The length of the distal root of #75 was greater in boys of 8 years old than in girls (*p* = 0.02)
- The length of the mesial root and the CRR- m of #74 was greater in 9-year-old boys than in girls (*p* < 0.05)
- The developmental stage of #34 was greater in boys than in 9-year-old girls (*p* = 0.006)
- The distal root length of #75 was greater in 10-year-old boys than in girls (*p* = 0.02).

The roots of the primary molar and its relationship with the germ of the successor were compared. In general, the differences were in developmental stages D and E of the first premolar (#34), in which the mesial root and the CRR-m of #74 were greater than those of the distal root. Due to the gender differences, we separated the analysis of the CRR-m and CRR-d differences in each stage of development by gender. The differences between roots in girls occurred in stage C and D (Table 1). In boys, the mesial root and the CRR-m of #75 were greater than the distal root and CRR-d, respectively, during developmental stage D of the second premolar (tooth #35) (Table 2). The correlation between chronological age and the state of development was studied, obtaining an almost perfect relationship between chronological age and the development stage of #34 (Pearson’s correlation coefficient r = 0.809) and #35 (r = 0.729). We were also able to establish a negative correlation between the stage of premolar formation and the CRRs of #74 and #75.

## 4. Discussion

Root resorption of primary teeth has been described by longitudinal studies [11,20], but that method required taking too many X-rays in children. Thus, we established a method that would measure the resorption of dental roots at a specific time (a cross-sectional study). Corono-radicular proportions were used to reduce the influence on linear measurements of phenomena, such as magnification, position of the head, and racial differences in tooth size. After reviewing the literature, we did not find any author who had previously used them.

Daito et al. studied this relationship in Japanese children and observed that when radicular resorption of the primary tooth begins, the premolar successor presents the crown fully formed and begins to form its root. We also observed that premolar root formation begins when the decrease of CRR becomes evident during Demirjian stage E [21].

As there is a relationship between the resorption of the root of primary teeth and the eruption of permanent teeth, the roots of the primary molars were not completely resorbed when the lower premolar successors were in Demirjian stage F, leaving a percentage of the root that is not resorbed which is less than the height of the crown (CRR <1). Complete root resorption of the primary molars occurred when the premolar developed two-thirds of its root, or when the apical closure had begun, which coincided with other studies [6,22].

Our results agree with other authors regarding the difference between sexes. Previous studies reported that root resorption and premolar formation are more advanced in girls than in boys, and that these differences are accentuated with age (differences in premolar formation were not significant in our study, except at #34) [11,13,23].

In our study, root resorption was always greater in the distal root, with significant differences when the premolar was in stages C–E. These findings agree with those obtained by Haavikko, [22,24], but they differ from the results obtained by Harris [25], although this author based his work on previous research carried out on lateral radiographs [20].

Our data suggest that, although there are no significant differences in CRR at any of the successor premolar formation stage, the successor premolar germ is more distal in the initial stages of formation, positioning itself centrally between the primary molar roots as its development progresses, in agreement with previous authors [14,15].

The study carried out presents some limitations that have been justified by previous studies on the subject. Orthopantomographies offer a global view of the structures of the lower third of the face, with less precision than intraoral radiographs [26] and a degree of distortion and magnification that varies depending on the X-ray device [27,28]; this distortion is greater in the horizontal plane [29]. However, the use of these radiographs has been shown to be valid for the determination of physiological resorption [30], the performance of vertical measurements [29,31,32], and the estimation of age in forensic medicine [33]. The lower left hemiarch was selected for the study, since according to various authors it is the one that suffers the least distortion [27,28,34,35,36,37] and magnification [31,36,37], and root superimposition is avoided. Physiological wear of primary teeth, which affects coronal length and is generalized in all children, was also taken into account [38], showing that the coronal height of teeth does not vary significantly with age according to our results in previous studies [39].

A weakness of the study is the heterogeneous age distribution of the sample, due to non-probabilistic sampling. This makes the interpretation of the data cautious in the extreme age data, especially in those of 11 and 12 years old, and more in accordance with children between 7 and 9 years old, who were the largest group. Another limitation is that some studies [40,41,42,43] establish the presence of great heterogeneity in dental size in different cultures or ethnic groups. However, studies of odontometry in primary teeth by Black in 1890 [16], Marseillier in 1937 [44], Kramer and Ireland in 1959 [45], and Liverside et al in 1993 [46] offer very similar data regarding the vertical dimensions of primary teeth. The meta-analysis carried out by Sujitha et al in 2022 [47] establishes the great presence of bias in odontometry studies, and the lack of evidence regarding tooth length. Due to this heterogeneity that is difficult to control, the ratio between the vertical measurement of the crown and the root was carried out using CRR in our study.

Despite the limitations, according to the results of our study and from the tables obtained, it is possible to determine the root length by means of the CRR through the age of the patient, allowing an approximation of the physiological resorption process to be established and, therefore, allowing for the elaboration of individualized therapeutic plans. Rowlands et al also state that premolar eruption can be predicted from CRR [48]. The current lines of research analyse the morphological changes of the pulp cells [49] and ultrastructural alterations in the pulp tissues [50] during the different stages of physiological root resorption, which will help to better understand this process.

## 5. Conclusions

Root resorption of the molar is slight and progressive when the premolar begins its formation until stage D, and began increasing at stage E. It is possible to determine the state of the child’s maturation and the CRR according to dental and chronological age. The mesial CRR is always greater than the distal CRR in all age groups. While the coronal length is stable as a function of age, the root length decreases with increasing age, being stable up to 6 years in the primary second molar. The results suggest that root resorption and premolar development is more advanced in girls than in boys for some age groups.

## Figures and Tables

**Table 1:** Comparison between the roots of #74 and its relationship with the germ of the successor.

Stage of Develpment #34	CRR-M #74	CRR-D #74	Differences			*t* Test *p*
	Mean ± S.D. ^+^	Mean ± S.D.	Mean Difference	Confidence Interval 95%		
				Higher	Lower	
Stage B	1.596 ± 0.113	1.502 ± 0.167	0.094	−0.018	0.206	0.081
Stage C	1.481 ± 0.203	1.406 ± 0.225	0.075	−0.009	0.158	0.078
Stage D	1.454 ± 0.262	1.326 ± 0.204	0.128	0.787	0.177	0.000 *
Stage E	1.178 ± 0.254	1.132 ± 0.246	0.046	−0.167	0.076	0.002 *
Stage F	0.890 ± 0.333	0.863 ± 0.308	0.027	−0.015	0.069	0.211
BOYS (N = 177)						
Stage B	1.557 ± 0.140	1.467 ± 0.196	0.090	−0.107	0.287	0.188
Stage C	1.470 ± 0.236	1.441 ± 0.264	0.028	−0.091	0.149	0.617
Stage D	1.470 ± 0.256	1.333 ± 0.200	0.057	0.014	0.101	0.001 *
Stage E	1.179 ± 0.260	1.121 ± 0.249	−0.009	−0.296	0.278	0.010 *
Stage F	0.924 ± 0.289	0.894 ± 0.273	0.030	−0.030	0.090	0.321
GIRLS (N = 145)						
Stage B	1.655 ± 0.21	1.555 ± 0.162	0.100	−1.170	1.371	0.500
Stage C	1.495 ± 0.157	1.360 ± 0.158	0.135	0.013	0.259	0.033 *
Stage D	1.435 ± 0.273	1.318 ± 0.211	0.117	0.054	0.179	0.001 *
Stage E	1.178 ± 0.247	1.145 ± 0.242	0.032	−0.007	0.728	0.106
Stage F	0.842 ± 0.387	0.820 ± 0.350	0.022	−0.038	0.082	0.455

^+^ S.D. Standard deviation. * *p* value ≤ 0.05.

**Table 2:** Comparison between the roots of the #75 and its relationship with the germ of the successor.

Stage of Development #35	CRR-m 75	CRR-d 75	Differences			*t* Test *p*
	Mean ± S.D. ^+^	Mean ± S.D.	Mean Difference	Confidence Interval 95%		
				Higher	Lower	
Stage A	1.520 ± 0.197	1.490 ± 0.214	0.030	−0.026	0.086	0.208
Stage B	1.476 ± 0.185	1.464 ± 0.161	0.011	−0.022	0.044	0.484
Stage C	1.420 ± 0.140	1.414 ± 0.179	0.006	−0.055	0.068	0.846
Stage D	1.320 ± 0.177	1.306 ± 0.172	0.013	−0.008	0.035	0.229
Stage E	1.201 ± 0.230	1.190 ± 0.216	0.011	−0.018	0.039	0.467
Stage F	0.931 ± 0.276	0.940 ± 0.267	−0.009	−0.04	0.030	0.642
BOYS (N = 221)						
Stage B	1.432 ± 0.187	1.409 ± 0.168	0.023	−0.014	0.059	0.195
Stage C	1.423 ± 0.137	1.469 ± 0.205	−0.046	−0.148	0.055	0.433
Stage D	1.305 ± 0.170	1.274 ± 0.152	0.032	0.001	0.062	0.043 *
Stage E	1.205 ± 0.239	1.207 ± 0.234	−0.002	−0.040	0.036	0.916
Stage F	0.953 ± 0.280	0.986 ± 0.283	−0.033	−0.083	0.016	0.180
GIRLS (N = 180)						
Stage A	1.490 ± 0.170	1.415 ± 0.205	0.075	−0.243	0.392	0.205
Stage B	1.519 ± 0.182	1.519 ± 0.141	0.000	−0.062	0.062	1.000
Stage C	1.417 ± 0.148	1.371 ± 0.148	0.047	−0.033	0.126	0.234
Stage D	1.335 ± 0.183	1.342 ± 0.185	−0.007	−0.038	0.023	0.633
Stage E	1.195 ± 0.217	1.165 ± 0.186	0.030	−0.014	0.075	0.173
Stage F	0.902 ± 0.271	0.879 ± 0.235	0.023	−0.042	0.088	0.474

^+^ S.D. Standard deviation. * *p* value ≤ 0.05.

## Data Availability

The data presented in this study are available on request from the corresponding author.

## Appendix A

**Table A1:** Crown-to-root ratio (CRR) of #74 and #75, classified according to chronological age.

Chronological Age Range	Root	n	CRR #74 ± S.D. ^+^	n	CRR #75 ± S.D.
**4/0–4/12**	**Mesial**	23	1.53 ± 0.15	23	1.52 ± 0.16
	**Distal**		1.42 ± 0.21		1.51 ± 0.18
**5/0–5/12**	**Mesial**	20	1.52 ± 0.31	24	1.39 ± 0.17
	**Distal**		1.39 ± 0.25		1.38 ± 0.17
**6/0–6/12**	**Mesial**	45	1.37 ± 0.23	52	1.37 ± 0.16
	**Distal**		1.27 ± 0.213		1.38 ± 0.17
**7/0–7/12**	**Mesial**	81	1.28 ± 0.29	95	1.31 ± 0.22
	**Distal**		1.21 ± 0.25		1.29 ± 0.20
**8/0–8/12**	**Mesial**	65	1.11 ± 0.27	81	1.21 ± 0.22
	**Distal**		1.07 ± 0.26		1.20 ± 0.20
**9/0–9/12**	**Mesial**	62	0.91 ± 0.30	82	1.06 ± 0.27
	**Distal**		0.88 ± 0.28		1.05 ± 0.26
**10/0–10/12**	**Mesial**	21	0.89 ± 0.39	33	0.97 ± 0.30
	**Distal**		0.89 ± 0.39		0.99 ± 0.29
**11/0–11/12**	**Mesial**	4	0.84 ± 0.37	8	0.98 ± 0.30
	**Distal**		0.76 ± 0.47		1.03 ± 0.33
**12/0–12/12**	**Mesial**	1	1.13 *	3	0.95 ± 0.26
	**Distal**		0.89 *		0.91 ± 0.20

^+^ S.D. Standard deviation. * We have not calculated the standard deviation due to the sample size.

**Table A2:** Crown-to-root ratio of #74 and #75, classified according to dental age.

Dental Age Range	Root	n	CRR #74 ± S.D. ^+^	n	CRR #75 ± S.D.
**4/0–4/12**	**Mesial**	8	1.60 ± 0.14	8	1.47 ± 0.12
	**Distal**		1.42 ± 0.20		1.46 ± 0.13
**5/0–5/12**	**Mesial**	14	1.50 ± 0.21	15	1.47 ± 0.21
	**Distal**		1.47 ± 0.25		1.45 ± 0.18
**6/0–6/12**	**Mesial**	16	1.51 ± 0.19	20	1.45 ± 0.16
	**Distal**		1.40 ± 0.2		1.43 ± 0.20
**7/0–7/12**	**Mesial**	108	1.36 ± 0.26	130	1.36 ± 0.17
	**Distal**		1.26 ± 0.22		1.35 ± 0.16
**8/0–8/12**	**Mesial**	94	1.12 ± 0.26	108	1.21 ± 0.19
	**Distal**		1.09 ± 0.24		1.19 ± 0.19
**9/0–9/12**	**Mesial**	46	0.93 ± 0.29	62	1.12 ± 0.30
	**Distal**		0.91 ± 0.31		1.11 ± 0.28
**10/0–10/12**	**Mesial**	23	0.81 ± 0.41	30	0.98 ± 0.28
	**Distal**		0.82 ± 0.36		0.97 ± 0.31
**11/0–11/12**	**Mesial**	11	0.73 ± 0.33	19	0.86 ± 0.19
	**Distal**		0.67 ± 0.32		0.98 ± 0.24
**12/0–12/12**	**Mesial**	2	1.07 ± 0.15	8	0.68 ± 0.19
	**Distal**		0.93 ± 0.04		0.73 ± 0.20

^+^ S.D. Standard deviation.

**Table A3:** Crown-to-root ratio of #74 and #75, classified according to the stage of development of the premolar successor.

Stages of Development of #34	Root	N	CRR #74 ± S.D. ^+^	Stages of Development of #35	ROOT	n	CRR #75 ± S.D.
**A**	**Mesial**	0	-	**A**	**MESIAL**	0	1.52 ± 0.20
	**Distal**		-		**DISTAL**		1.49 ± 0.22
**B**	**Mesial**	5	1.60 ± 0.21	**B**	**MESIAL**	22	1.48 ± 0.19
	**Distal**		1.50 ± 0.17		**DISTAL**		1.46 ± 0.16
**C**	Mesial	28	1.48 ± 0.20	**C**	MESIAL	32	1.42 ± 0.14
	**Distal**		1.41 ± 0.23		**DISTAL**		1.41 ± 0.18
**D**	**Mesial**	61	1.45 ± 0.26	**D**	**MESIAL**	139	1.32 ± 0.18
	**Distal**		1.33 ± 0.20		**DISTAL**		1.31 ± 0.17
**E**	**Mesial**	147	1.18 ± 0.25	**E**	**MESIAL**	5	1.20 ± 0.23
	**Distal**		1.13 ± 0.25		**DISTAL**		1.19 ± 0.21
**F**	**Mesial**	79	0.89 ± 0.33	**F**	**MESIAL**	70	0.93 ± 0.28
	**Distal**		0.86 ± 0.31		**DISTAL**		0.94 ± 0.27
**G**	**Mesial**	2	0.25 ± 0.28	**G**	**MESIAL**	2	0.98 ± 0.12
	**Distal**		0.25 ± 0.28		**DISTAL**		1.11 ± 0.07
**H**	**Mesial**	0	-	**H**	**MESIAL**	0	-
	**Distal**		-		**DISTAL**		-

^+^ S.D. Standard deviation.
